# Relationship between plasma asprosin, dry matter intake, and plasma glucose at different stages of lactation

**DOI:** 10.3389/fvets.2025.1588671

**Published:** 2025-05-21

**Authors:** Yinyin Chen, Maocheng Jiang, Yan Li, Yanjing Su, Siwei Luo, Peng Wu, Guoqi Zhao, Miao Lin, Kang Zhan

**Affiliations:** ^1^Department of Animal Husbandry and Veterinary Medicine, Jiangsu Polytechnic College of Agriculture and Forestry, Jurong, China; ^2^College of Animal Science and Technology, Yangzhou University, Yangzhou, China; ^3^Supervision and Testing Center for Agricultural Product Quality, Yancheng, China; ^4^Bright Farming Co., Ltd., Key Laboratory of Dairy Cattles Genetic Improvement in Southern China Ministry of Agriculture and Rural Affairs, Shanghai, China; ^5^Institute of Grassland Research, Chinese Academy of Agricultural Sciences, Hohhot, China; ^6^Hangzhou King Techina Feed Co., Ltd, Hangzhou, China

**Keywords:** asprosin, dry matter intake, hepatocytes, gluconeogenesis, glucose, postpartum

## Abstract

In postpartum dairy cows, dry matter intake (DMI) decreases dramatically, resulting in reduced glucose production and negative energy balance (NEB). Asprosin is a centrally acting orexigenic protein hormone secreted by adipose tissue, and it promotes glucose production in the liver. However, the effects of asprosin on hepatic glucose output in primary bovine hepatocytes, as well as the relationship between plasma asprosin and dry matter intake at different stages of lactation, have not yet been reported. Our results demonstrated that fibrillin 1 (FBN1) exhibited significantly higher mRNA expression in the mammary gland and adipose tissue. The bovine His-asprosin proteins were > 90% pure, as confirmed by Coomassie Blue-stained SDS-PAGE gel analysis. Asprosin increased (*p* = 0.031) the mRNA expression of phosphoenolpyruvate carboxykinase 2 (PCK2) in primary bovine hepatocytes compared to the control group. Remarkably, glucose output (*p* = 0.03) in the primary bovine hepatocytes exposed to asprosin was higher than that in the control group. In addition, asprosin was found to promote PKA activity in primary bovine hepatocytes. The postpartum dairy cows exhibited significantly lower plasma asprosin levels compared to those at 110 and 230 days relative to parturition (*p* < 0.01). Notably, plasma asprosin levels were positively correlated with DMI at different stages of lactation. These findings indicate that increased levels of circulating asprosin should be considered a novel resolution strategy for improving DMI and addressing negative nutrient balance during the postpartum period.

## 1 Introduction

During the transition to the lactation period, dairy cows experience a dramatic decrease in dry matter intake (DMI), triggering an attenuation in glucose production, a negative energy balance (NEB), and a negative nutrient balance (1, 2). Subcutaneous adipose tissue mobilization is enhanced, while lipogenesis in adipocytes is reduced due to decreased circulating plasma insulin and increased concentrations of circulating catecholamines, growth hormone, and glucocorticoids in postpartum dairy cows (3). In addition, glucose uptake and utilization by adipose tissue is reduced during the early lactation stage; however, they recover as the dairy cow progresses into mid-lactation (4). This suggests that adipose tissues show a metabolic syndrome process in postpartum dairy cows. In the current study, a major challenge identified in the management of postpartum dairy cows is achieving the adequate increase in appetite, DMI, and glucose production while maintaining energy homeostasis.

Asprosin, which is a C-terminal cleavage product of fibrillin 1 (FBN1) generated by the furin protease, is a centrally acting orexigenic protein hormone secreted by adipose tissue (5, 6). Circulating asprosin travels to the liver and binds to the G protein-coupled receptor OLFR734 to enhance gluconeogenesis and hepatic glucose production through the activation of the cAMP-PKA signaling pathway (7, 8). However, Olfr734 knockout mice did not show a reduction in appetite or body weight (8). A previous study reported that circulating asprosin crosses the blood–brain barrier to inhibit anorexigenic proopiomelanocortin (POMC) neurons and activate orexigenic AgRP neurons to stimulate appetite behaviors (6). A recent study demonstrated that protein tyrosine phosphatase receptor D (Ptprd) serves as the orexigenic receptor for asprosin, contributing to an increase in appetite and body weight in AgRP neurons (9). In addition, asprosin promotes the expression of gluconeogenic genes, such as G6PC and PCK1, and enhances glucose production (7). In ruminants, gluconeogenesis, which mainly occurs in the liver, is one of the most important mechanisms for meeting glucose requirements (10). However, the mechanisms underlying the regulatory effects of asprosin on gluconeogenic gene expression and glucose output in primary bovine hepatocytes, as well as the relationship between plasma asprosin and DMI at different stages of lactation, remain unknown. We hypothesized that dairy cows in the post-perinatal period exhibited a low plasma asprosin level because of the substantial reduction in DMI and glucose production.

Therefore, the objectives of this study were as follows: First, to test the possibility that plasma asprosin levels are reduced in postpartum dairy cows, we determined whether and where FBN1 and furin are expressed in bovine tissues, as well as whether asprosin is present in circulating blood. Second, this study aimed to evaluate the effect of asprosin on glucose production in bovine hepatocytes, investigate the concentration of plasma asprosin, and explore the relationship between plasma asprosin and DMI at different stages of lactation. The findings may provide a novel strategy to increase DMI, enhance glucose production, and maintain relative energy balance in postpartum dairy cows.

## 2 Materials and methods

The animal study was reviewed and approved in accordance with the principles of Yangzhou University and by the Institutional Animal Care and Use Committee (SYXK (Su) IACUC 2012-0029). Written informed consent was obtained from the owners for the participation of their animals in this study. Six Holstein cows from the experimental farm of Yangzhou University were used to determine the expression of FBN1 and furin in different tissues. Early lactation (0 and 21 days after parturition, *n* = 30), mid-lactation (*n* = 30), and late-lactation (*n* = 30) Holstein cows were used to detect circulating asprosin in the blood. These cows were fed a total mixed ration (TMR) to meet 100% of the (National Research Council) NRC requirements. The cows were milked three times daily at 8:00, 14:00, and 21:00. Blood samples (approximately 5 mL) were collected, and the DMI was also recorded. Plasma samples were immediately transported to the laboratory and stored at −80°C until the further analysis of asprosin and glucose levels.

The cDNA of bovine FBN1 (2732-2871 amino acids) was cloned and subsequently sub-cloned into a pET-30a vector using Nde I and Hind III for expression in *E. coli*. The *E. coli* strain used was BL21(DE3), obtained from GenScript company. The fusion protein expressed in *E. coli* consisted of 147 amino acids, including an amino-acid Met, a six-amino-acid His tag at the N terminus, and a 140-amino-acid wild-type asprosin. The pET-30a vector containing the 140-amino-acid asprosin was transformed, and the plate was incubated upside down at 37°C overnight. Cell pellets were resuspended in a lysis buffer, followed by sonication. The precipitate was dissolved using a denaturing imidazole buffer. The target protein was obtained through two-step purification using a Ni column and a Superdex 200 column. The concentration was determined using the Bradford protein assay with BSA as the standard. Protein purity and molecular weight were determined by standard SDS-PAGE and Western blot analyses.

Bovine hepatocytes were obtained as previously described, using the collagenase IV (Invitrogen, Shanghai, China) perfusion liver tissues collected from three non-lactating, non-gestating dairy cows at a local abattoir (11). The hepatocytes were resuspended in a DMEM/F12 medium supplemented with 10% FBS, 1 μM insulin, 1 μM dexamethasone, 100 U/mL penicillin, and 100 μg/mL streptomycin (Solarbio, Beijing, China). To investigate the expression of genes involved in the gluconeogenic pathway in primary bovine hepatocytes exposed to asprosin, the hepatocytes (2 × 10^5^ cells/well) were seeded in 6-well plates and were grown at 37°C with 5% CO_2_. The cells were divided into two experimental groups: 1. Control group, DMEM/F12 medium and 2. asprosin treatment group, DMEM/F12 medium containing 100 nmol/L asprosin. All samples were incubated for 6 h. After the incubation, total RNA was isolated from the cultured cells using a TRIzol kit (Tiangen, Beijing, China). Reverse transcription (RT) was performed using an RT kit (Takara, Beijing, China). Before performing qRT-PCR on the samples, the amplification efficiencies of all primers were determined using a standard dilution series. The primers listed in Table 1 are from a previous study (12). The relative expression of target genes was normalized to the geometric mean of the selected reference genes, GAPDH, and was calculated using the 2^−ΔΔCT^ method. For the measurement of glucose output in the primary bovine hepatocytes exposed to asprosin, the medium was then replaced with 1 mL of glucose-free DMEM and supplemented with 10 mM lactate and 1 mM sodium pyruvate. After incubation for an additional 2 h, the glucose level in the medium was determined using a kit (Applygen, E1011, Beijing, China). The results were normalized to protein content.

**Table 1:** Primers for real-time PCR analysis.

Gene	Primer sequence, 5′ to 3’	Accession number	Source
PCK2	F: 5 TGACTGGGCAAGGGGAGCCG 3 * R: 5 GGGGCCACCCCAAAGAAGCC 3	NM_001205594.1	Zhan et al. (18)
PC	F: 5 CCACGAGTTCTCCAACACCT 3 R: 5 TTCTCCTCCAGCTCCTCGTA 3	NM_177946.4	Zhan et al. (18)
G6PC	F: 5TGATGGACCAAGAAAGATCCAGG 3 R: 5TATGGATTGACCTCACTGGCCCTCTT 3	NM_001076124.2	Zhan et al. (18)
FBP1	F: 5 ATAGAGAAGGCAGGAGGAAT 3 R: 5 CAGGAACTCAGTCACATCTT 3	NM_001034447	Zhan et al. (18)
GAPDH	F: 5 GGGTCATCATCTCTGCACCT 3 R: 5 GGTCATAAGTCCCTCCACGA 3	NM_001034034	Zhan et al. (18)
FBN1	F: 5 AGACAGCCGTGTTCGCTTTC 3 R: 5 AAGTGGAGGTAGCTGATCCC 3	NM_174053.2	This study
Furin	F: 5 GCATCGAGAAGAACCACCCA 3 R: 5 CTCCACGGCATCTGTCACTT 3	NM_174136	This study

*F, forward; R, reverse.

The cells were lysed to extract total protein using a RIPA lysis and extraction buffer (Thermo Scientific, Shanghai, China) containing 1 × protease inhibitor cocktail (Thermo Scientific) and 1 × phosphatase inhibitor cocktail tablets (Roche, Shanghai, China). Protein concentrations were determined using a BCA kit (Beyotime, Beijing, China). Equal amounts of protein lysates were fractionated by SDS-PAGE and transferred to nitrocellulose membranes (PALL, Shanghai, China). The following primary antibodies and goat anti-rabbit IgG were obtained from Cell Signaling Technology (CST, Shanghai, China): GAPDH and phosphorylated (p)-PKA (CST, Shanghai, China). Target bands were detected using the SuperSignal West Femto Maximum Sensitivity Substrate (Thermo Scientific).

### 2.1 Statistical analysis

Statistical analysis was performed using an independent samples *t*-test with SPSS 19.0 software (SPSS Inc.; Chicago, IL, United States). A *p*-value of < 0.05 was considered significant, and a *p*-value of < 0.01 was considered highly significant. Trends toward significance were discussed at 0.05 < *p* < 0.10.

## 3 Results

### 3.1 Expression of FBN1 and furin mRNA in different bovine tissues

**Figure 1:**
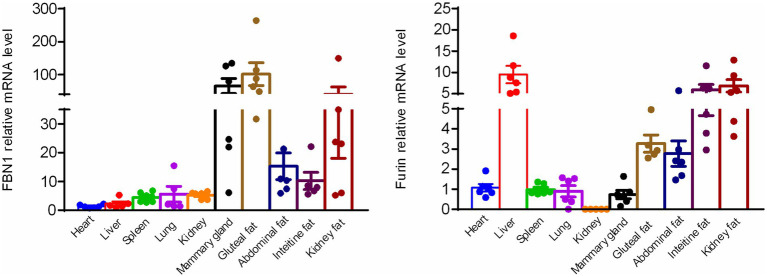
Expression of FBN1 and furin mRNA in various bovine tissues, as determined by qRT-PCR (*n* = 6).

To confirm the expression of FBN1 and the furin protease, the mRNA expression profiles of FBN1 and the furin protease were assessed across various metabolically important organs in bovine tissues. Based on the qRT-PCR analysis, both FBN1 and furin mRNA were expressed in various bovine tissues, including the heart, liver, spleen, lung, kidney, mammary gland, haunch fat, abdominal fat, intestinal fat, and renal fat. The FBN1 showed much higher mRNA expression in the mammary gland and adipose tissue compared to the heart, liver, spleen, lung, and kidney. In addition, the liver tissue displayed the highest furin mRNA expression. The white adipose tissue demonstrated significantly higher mRNA expression than the heart, spleen, lung, and kidney (Figure 1). These results indicate that the white adipose tissue and mammary gland may generate and secrete asprosin due to the significantly higher expression levels of FBN1.

### 3.2 Expression evaluation of recombinant asprosin

**Figure 2:**
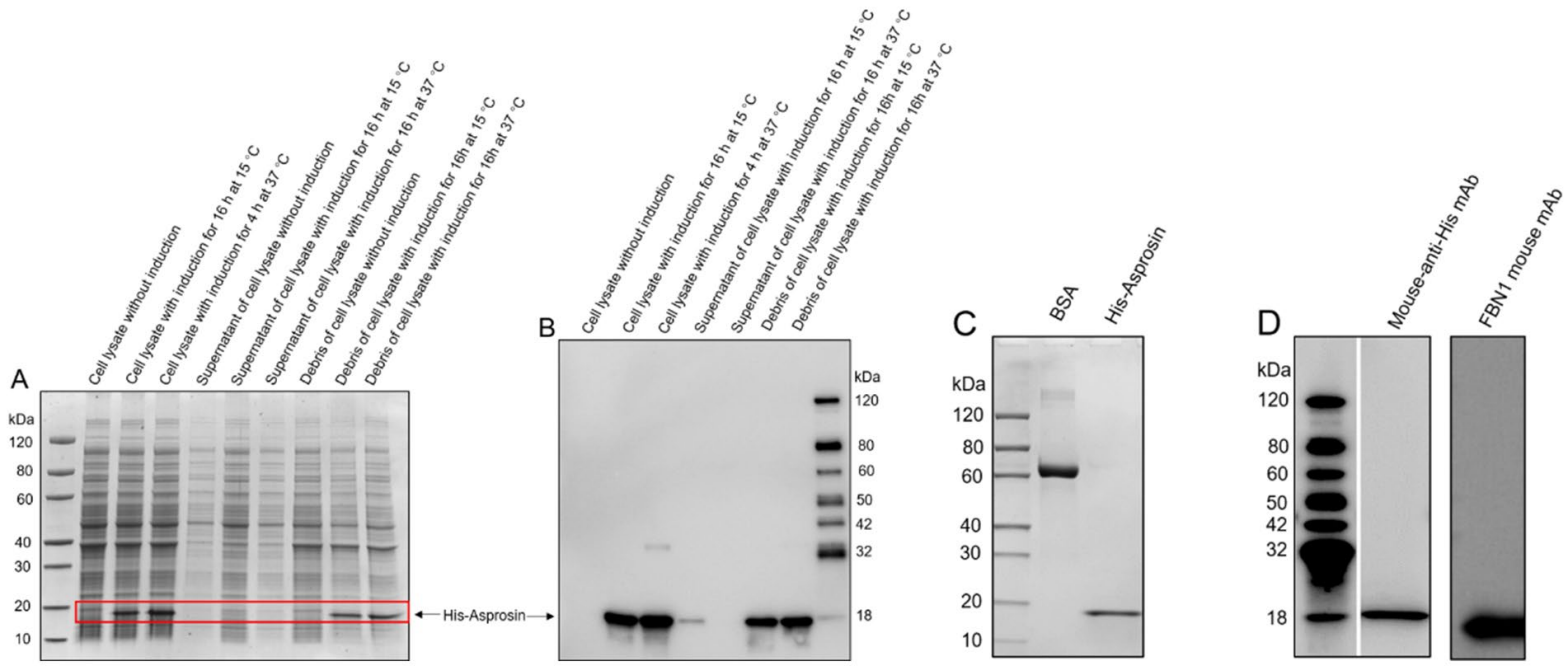
Expression evaluation of bovine recombinant asprosin and scale-up expression. **(A)** SDS-PAGE analysis of asprosin cloned into pET-30a and expressed in the BL21 (DE3) strain under different conditions. **(B)** Western blot analysis of the protein lysate sample under different treatment conditions using an anti-His antibody. **(C)** SDS-PAGE analysis of purified bovine recombinant asprosin from scale-up expression. **(D)** Western blot analysis of purified bovine recombinant asprosin using a mouse anti-His antibody and a mouse anti-FBN1 monoclonal antibody (against 2772–2871 amino acids immunogen).

To investigate the circulating asprosin level in bovine plasma, bovine FBN1 (2732-2871 amino acids) was sub-cloned into a pET-30a vector for expression in *E. coli* in order to obtain bovine recombinant asprosin. Our results revealed that recombinant asprosin was scarcely found in the supernatant of the cell lysate but was expressed in the cell lysate, as indicated by SDS-PAGE analysis (Figure 2A). In addition, His-tagged recombinant asprosin was detected in the cell lysate using an anti-His antibody by Western blotting, with only a slight expression in the supernatant of the cell lysate (Figure 2B). This suggests that bovine recombinant asprosin was present in the inclusion bodies of *E. coli*. The purified bovine recombinant asprosin was found in the scale-up expression, as indicated by SDS-PAGE (Figure 2C). Moreover, the purified bovine recombinant asprosin was detected using a mouse anti-His antibody and a mouse anti-FBN1 monoclonal antibody (against 2772-2871 amino acids immunogen) via Western blotting (Figure 2D). The purity level of the purified bovine recombinant asprosin was estimated using Coomassie Blue-stained SDS-PAGE gel analysis under reducing conditions. The bovine His-asprosin proteins used in all recombinant protein experiments were > 90% pure.

### 3.3 Effects of asprosin on the expression of gluconeogenic genes and glucose production

**Figure 3:**
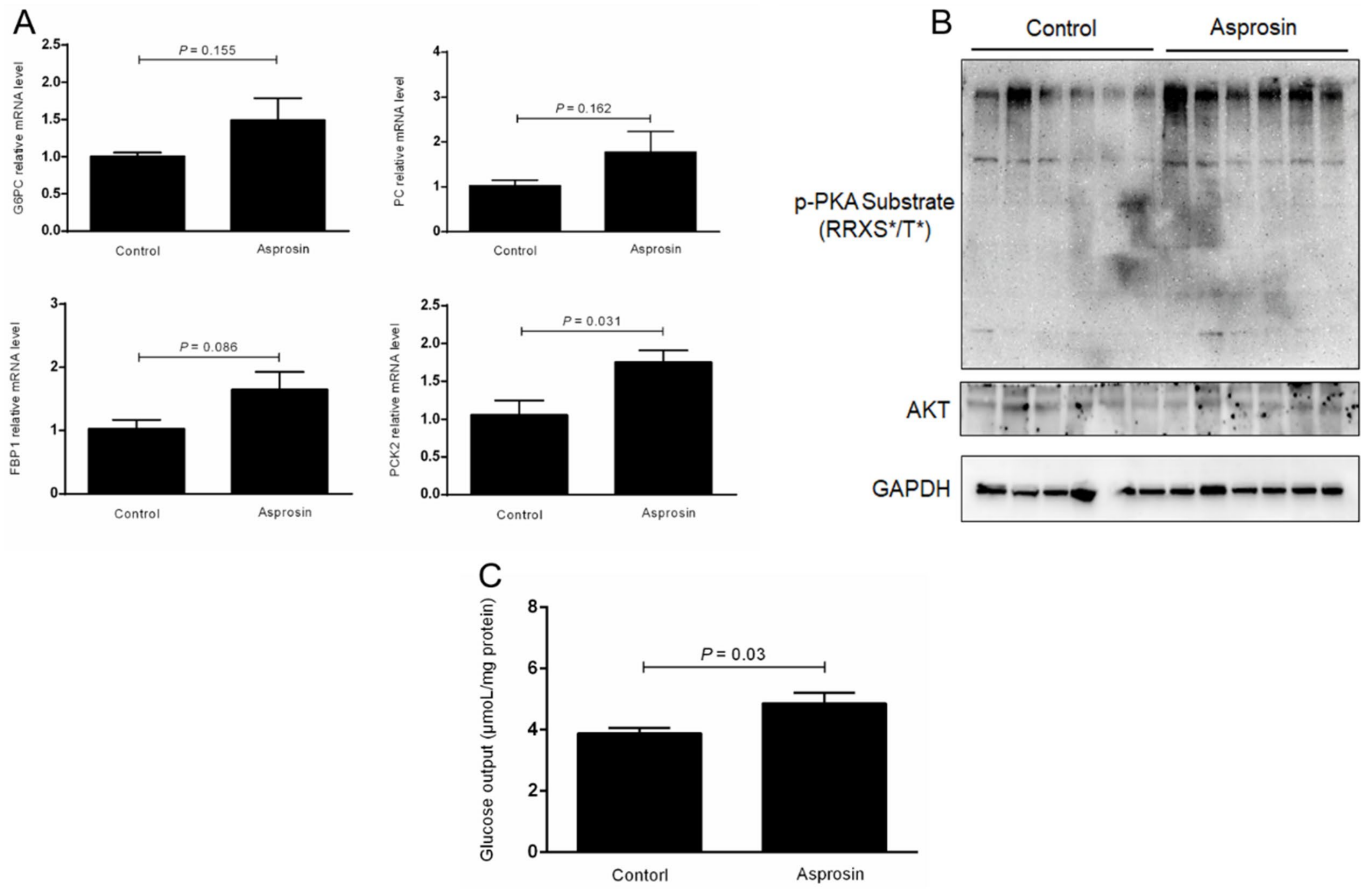
Asprosin enhanced glucose production via the activation of p-PKA in primary bovine hepatocytes. **(A)** qRT-PCR analysis of the expression of gluconeogenic genes (*n* = 4). **(B)** Western blot analysis of PKA activity using immunoblotting with a PKA substrate antibody (*n* = 6). **(C)** Effects of asprosin on glucose output in primary bovine hepatocytes (*n* = 6).

To assess whether asprosin can promote the mRNA expression of genes involved in the gluconeogenesis pathway and glucose production, these genes were investigated using qRT-PCR, and hepatic glucose release into the medium was analyzed using a kit. Our results demonstrated that the mRNA expression of glucose-6-phosphatase (G6PC) and pyruvate carboxylase (PC) was elevated in the bovine hepatocytes treated with asprosin compared to the control group, although no significant difference was observed (Figure 3A). Remarkably, asprosin enhanced (*p* < 0.05) the mRNA expression of phosphoenolpyruvate carboxykinase 2 (PCK2) in primary bovine hepatocytes compared to the control group (Figure 3A). In addition, fructose-1,6-bisphophatase (FBP1) tended to be upregulated (*p* = 0.086) in primary bovine hepatocytes treated with asprosin compared to the control group (Figure 3A). To demonstrate whether asprosin can activate the PKA signal pathway, PKA activity was evaluated by immunoblotting with a PKA substrate antibody in the primary bovine hepatocytes. Our results showed that PKA activity was significantly elevated upon asprosin stimulation, as determined by immunoblotting with a PKA substrate antibody, although no significant change was observed in AKT activity (Figure 3B). Importantly, hepatic glucose release into the medium was significantly increased (*p* < 0.05) in the primary bovine hepatocytes exposed to asprosin compared to the control group (Figure 3C).

### 3.4 Plasma levels of asprosin at different stages of lactation

**Figure 4:**
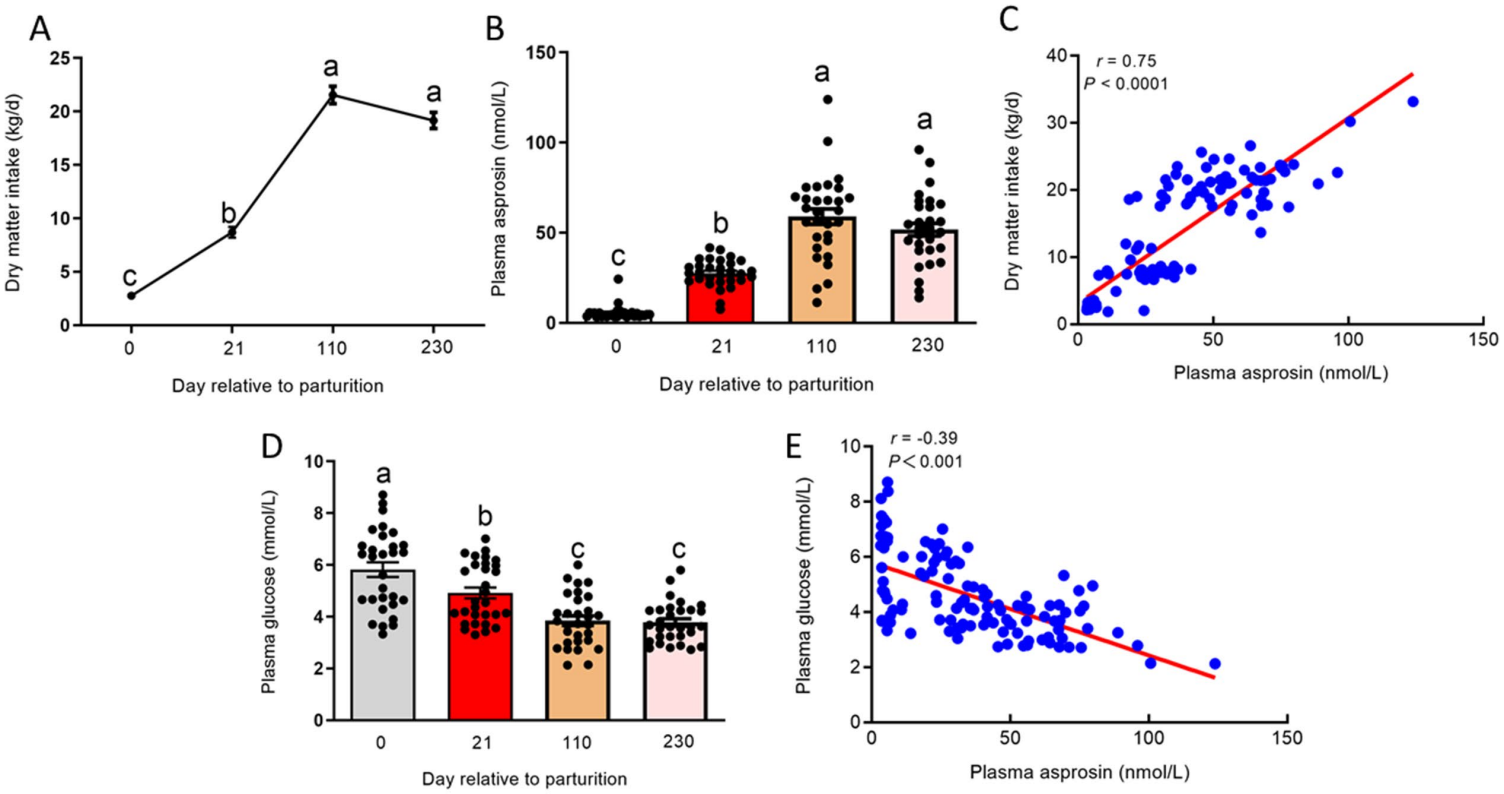
Plasma levels of asprosin at different stages of lactation dairy cows. **(A)** DMI at 0, 21, 110, and 230 days relative to parturition. **(B)** Sandwich ELISA was used to detect plasma asprosin levels at 0, 21, 110, and 230 days relative to parturition. **(C)** Relationship between plasma asprosin and DMI at 0, 21, 110, and 230 days relative to parturition. **(D)** The concentration of plasma glucose at 0, 21, 110, and 230 days relative to parturition. **(E)** Relationship between plasma asprosin and glucose at 0, 21, 110, and 230 days relative to parturition.

In postpartum cows, DMI was dramatically decreased, triggering an attenuation in glucose production. Our results showed that DMI was significantly reduced (*p* < 0.01) at 0 and 21 days relative to parturition compared to 110 and 230 days relative to parturition (Figure 4A). The decreased DMI may have enhanced the circulating asprosin level because asprosin is a centrally acting orexigenic protein hormone. To investigate circulating asprosin levels at different stages of lactation in dairy cows, the plasma levels of asprosin should be investigated. To measure circulating asprosin levels, we developed a sandwich ELISA. Asprosin was observed to be present in bovine plasma at consistent nanomolar levels (Figure 4B), which is consistent with previous studies (5, 13). At 0 day relative to parturition, circulating asprosin levels were lower, but plasma asprosin levels were markedly elevated (*p* < 0.01) at 21 days relative to parturition. Interestingly, plasma asprosin levels were further increased at 110 and 230 days relative to parturition (Figure 4B). In addition, plasma asprosin levels were positively correlated (*p* < 0.01) with DMI across different stages of lactation (Figure 4C). In contrast, the postpartum dairy cows displayed (*p* < 0.01) significantly higher plasma glucose levels compared to those at 110 and 230 days relative to parturition (Figure 4D), and plasma asprosin levels were negatively correlated (*p* < 0.01) with plasma glucose levels across different stages of lactation (Figure 4E). It is worth noting that plasma glucose levels were higher at 0 and 21 days relative to parturition because glucose uptake and utilization by adipose tissue, muscles, and other peripheral tissues are reduced in postpartum dairy cows. However, the net production of glucose is also limited in postpartum dairy cows.

## 4 Discussion

Asprosin, a C-terminal cleavage product of FBN1 generated by furin protease, is a centrally acting orexigenic protein hormone secreted by adipose tissue (5). Asprosin promotes hepatic glucose production (5, 7) and crosses the blood–brain barrier to activate orexigenic AgRP neurons to stimulate feeding behaviors in the hypothalamus (6, 8). It is well known that DMI is dramatically decreased in postpartum dairy cows, leading to reduced glucose production. We hypothesized that dairy cows in the post-perinatal period may exhibit a low plasma asprosin level due to substantial reduction in DMI and glucose production. However, circulating asprosin levels at different stages of lactation remain unknown. Therefore, bovine recombinant asprosin should first be obtained through a prokaryotic expression system *in vitro*.

To determine whether bovine can secret asprosin, we first detected the expression of FBN1 and the furin protease in various metabolically important organ tissues. The bovine mammary gland and adipose tissues displayed significantly higher FBN1 expression levels compared to the heart, liver, spleen, lung, and kidney. The furin protease exhibited the highest expression in bovine liver tissues, and white adipose tissue demonstrated much higher mRNA expression compared to the heart, spleen, lung, mammary gland, and kidney. A previous study also demonstrated that white adipose tissue is one of the sources of circulating plasma asprosin (5). It is well known that adipose tissue acts as an endocrine organ and a regulator of energy homeostasis, which is consistent with the secretion of asprosin by adipose tissue.

Asprosin functions to increase plasma glucose concentrations (5). Gluconeogenesis, which mainly occurs in the liver, is one of the most important mechanisms for meeting glucose requirements in postpartum cows. The rate of gluconeogenesis in the liver is controlled by the activity of several flux-controlling enzymes, including PCK, G6PC, and PC, that are responsive to hormonal and allosteric regulation (14, 15). A previous study indicated that hepatic PCK1 mRNA expression in dairy cows increased when DMI was elevated during early lactation (10).

To investigate whether asprosin affects the expression of genes involved in hepatic gluconeogenesis, we detected the key expression of genes in primary bovine hepatocytes, including four rate-limiting enzymes G6PC, FBP1, PC, and PCK (16). We found that asprosin enhanced the mRNA expression of PCK2 in bovine hepatocytes compared to the control group. In addition, FBP1 tended to be upregulated in the bovine hepatocytes treated with asprosin. More importantly, glucose output was increased in the bovine hepatocytes exposed to asprosin compared to the control group. Previous studies mainly focused on the effects of propionate-induced gluconeogenesis (17, 18). The proportion of propionate involved in gluconeogenesis is between 50 and 60% in bovine hepatocytes (14, 19). In the present study, asprosin was found to have a significant effect on the expression of genes involved in gluconeogenesis and glucose output in primary bovine hepatocytes, suggesting that asprosin may respond to different gluconeogenic precursors to promote hepatic glucose production. Asprosin activates the OLFR734 receptor to induce glucose output via the cAMP-PKA second-messenger pathway (7). Our results showed that PKA activity was elevated in bovine hepatocytes induced by asprosin; however, AKT activity was not altered. These findings demonstrate that asprosin induces glucose output via the PKA second-messenger pathway in bovine hepatocytes.

Circulating asprosin can cross the blood–brain barrier to inhibit POMC neurons and activate orexigenic AgRP neurons in order to induce feeding behaviors (6). Moreover, neutralization of asprosin by a monoclonal antibody in the blood leads to reduced feeding and body weight in obese mice (6, 20). The DMI was dramatically decreased in postpartum dairy cows. We hypothesized that dairy cows have a low level of plasma asprosin at 0 and 21 days relative to parturition. To investigate this, we selected dairy cows at 0, 21, 110, and 230 days relative to parturition to measure their circulating asprosin levels. We found that the cows at 0 day relative to parturition had lower circulating asprosin levels, but plasma asprosin levels were elevated at 21 days relative to parturition. Remarkably, plasma asprosin levels were further increased at 110 and 230 days relative to parturition. Conversely, the dairy cows displayed higher plasma glucose levels at 0 and 21 days relative to parturition. A previous study demonstrated that plasma asprosin levels are decreased by feeding due to a high plasma glucose condition, with high plasma glucose levels acting as a suppressor of plasma asprosin levels through a negative feedback loop (5). Although the DMI was dramatically reduced in postpartum dairy cows, plasma glucose levels still remained high because glucose uptake and utilization by adipose tissue, muscles, and other peripheral tissues are reduced in postpartum dairy cows (3). Therefore, high plasma glucose levels in postpartum dairy cows may inhibit asprosin secretion from adipose tissue. This finding aligns with our data showing high plasma glucose levels and low plasma asprosin levels in these cows. In addition, plasma asprosin levels are negatively correlated with plasma glucose levels at different stages of lactation. However, plasma asprosin levels are positively correlated with DMI at different stages of lactation. A major challenge in the management of postpartum dairy cows is increasing DMI sufficiently to maintain energy homeostasis. Asprosin secreted by adipose tissue activates orexigenic AgRP neurons to promote feeding behaviors. Bovine recombinant asprosin should be obtained and injected into the bloodstream to activate orexigenic AgRP neurons to increase DMI in postpartum dairy cows because plasma asprosin levels are positively correlated with DMI at different stages of lactation. In addition, the mechanisms underlying the regulatory effects of asprosin secreted by adipose tissue should be further investigated in postpartum dairy cows. Therefore, asprosin should be further considered for use as a novel resolution strategy to enhance DMI and improve energy homeostasis in dairy cows during the postpartum period.

In conclusion, postpartum cows exhibited significantly lower levels of asprosin compared to those at 110 and 230 days relative to parturition, suggesting that increased levels of circulating asprosin may promote DMI and glucose production. Therefore, asprosin should be further considered for use as a novel resolution strategy to enhance DMI and improve energy homeostasis during the postpartum period, which is critically important for milk production performance during both early and overall lactation periods.

## Data Availability

The original contributions presented in the study are included in the article/supplementary material, further inquiries can be directed to the corresponding author.

## References

[1] Contreras GA Strieder-Barboza C De Koster J. Symposium review: modulating adipose tissue lipolysis and remodeling to improve immune function during the transition period and early lactation of dairy cows. J Dairy Sci. (2018) 101:2737–52. doi: 10.3168/jds.2017-13340, PMID: 29102145

[2] Zhang Q Su H Wang F Cao Z Li S. Effects of energy density in close-up diets and postpartum supplementation of extruded full-fat soybean on lactation performance and metabolic and hormonal status of dairy cows. J Dairy Sci. (2015) 98:7115–30. doi: 10.3168/jds.2014-9112, PMID: 26254529

[3] Bauman DE Currie WB. Partitioning of nutrients during pregnancy and lactation: a review of mechanisms involving homeostasis and homeorhesis. J Dairy Sci. (1980) 63:1514–29. doi: 10.3168/jds.S0022-0302(80)83111-0, PMID: 7000867

[4] Bell AW Bauman DE. Adaptations of glucose metabolism during pregnancy and lactation. J Mammary Gland Biol Neoplasia. (1997) 2:265–78. doi: 10.1023/a:1026336505343 10882310

[5] Romere C Duerrschmid C Bournat J Constable P Jain M Xia F. Asprosin, a fasting-induced glucogenic protein hormone. Cell. (2016) 165:566–79. doi: 10.1016/j.cell.2016.02.063, PMID: 27087445 PMC4852710

[6] Duerrschmid C He Y Wang C Li C Bournat JC Romere C. Asprosin is a centrally acting orexigenic hormone. Nat Med. (2017) 23:1444–53. doi: 10.1038/nm.4432, PMID: 29106398 PMC5720914

[7] Li E Shan H Chen L Long A Zhang Y Liu Y. OLFR734 mediates glucose metabolism as a receptor of asprosin. Cell Metab. (2019) 30:319–328.e8. e318. doi: 10.1016/j.cmet.2019.05.022, PMID: 31230984

[8] Liu Y Long A Chen L Jia L Wang Y. The Asprosin–OLFR734 module regulates appetitive behaviors. Cell Discov. (2020) 6:19–3. doi: 10.1038/s41421-020-0152-4, PMID: 32337066 PMC7154029

[9] Mishra I Xie WR Bournat JC He Y Wang C Silva ES. Protein tyrosine phosphatase receptor δ serves as the orexigenic asprosin receptor. Cell Metab. (2022) 34:549–563.e8. doi: 10.1016/j.cmet.2022.02.012, PMID: 35298903 PMC8986618

[10] Greenfield R Cecava M Donkin S. Changes in mRNA expression for gluconeogenic enzymes in liver of dairy cattle during the transition to lactation. J Dairy Sci. (2000) 83:1228–36. doi: 10.3168/jds.S0022-0302(00)74989-7, PMID: 10877388

[11] Yang T Ma X Jiang M Cheng Z Datsomor O Zhao G. The role of tea tree oil in alleviating palmitic acid-induced lipid accumulation in bovine hepatocytes. Front Vet Sci. (2021) 8:814840. doi: 10.3389/fvets.2021.814840 35127885 PMC8814581

[12] Zhang Q Koser SL Donkin SS. Propionate induces mRNA expression of gluconeogenic genes in bovine calf hepatocytes. J Dairy Sci. (2016) 99:3908–15. doi: 10.3168/jds.2015-10312, PMID: 26947295

[13] Wang C-Y Lin T-A Liu K-H Liao C-H Liu Y-Y Wu VC-C. Serum asprosin levels and bariatric surgery outcomes in obese adults. Int J Obes. (2019) 43:1019–25. doi: 10.1038/s41366-018-0248-1, PMID: 30459402

[14] Aschenbach JR Kristensen NB Donkin SS Hammon HM Penner GB. Gluconeogenesis in dairy cows: the secret of making sweet Milk from sour dough. IUBMB Life. (2010) 62:869–77. doi: 10.1002/iub.400, PMID: 21171012

[15] Pilkis SJ Granner DK. Molecular physiology of the regulation of hepatic gluconeogenesis and glycolysis. Annu Rev Physiol. (1992) 54:885–909. doi: 10.1146/annurev.ph.54.030192.004321, PMID: 1562196

[16] Lin HV Accili D. Hormonal regulation of hepatic glucose production in health and disease. Cell Metab. (2011) 14:9–19. doi: 10.1016/j.cmet.2011.06.003, PMID: 21723500 PMC3131084

[17] Liu L Xing D Du X Peng T McFadden JW Wen L. Sirtuin 3 improves fatty acid metabolism in response to high nonesterified fatty acids in calf hepatocytes by modulating gene expression. J Dairy Sci. (2020) 103:6557–68. doi: 10.3168/jds.2019-17670 32331890

[18] Zhan K Yang TY Chen Y Jiang MC Zhao GQ. Propionate enhances the expression of key genes involved in the gluconeogenic pathway in bovine intestinal epithelial cells. J Dairy Sci. (2020) 103:5514–24. doi: 10.3168/jds.2019-17309, PMID: 32278554

[19] Huntington G. Energy metabolism in the digestive tract and liver of cattle: influence of physiological state and nutrition. Reprod Nutr Dev. (1990) 30:35–47. doi: 10.1051/rnd:19900103, PMID: 2184823

[20] Mishra I Duerrschmid C Ku Z He Y Xie W Silva ES. Asprosin-neutralizing antibodies as a treatment for metabolic syndrome. eLife. (2021) 10:e63784. doi: 10.7554/eLife.63784, PMID: 33904407 PMC8102062

